# Study on starch content detection and visualization of potato based on hyperspectral imaging

**DOI:** 10.1002/fsn3.2415

**Published:** 2021-06-22

**Authors:** Fuxiang Wang, Chunguang Wang, Shiyong Song, Shengshi Xie, Feilong Kang

**Affiliations:** ^1^ Inner Mongolia Agriculture University Hohhot China

**Keywords:** hyperspectral imaging, potato, starch, three sampling sites, visualization

## Abstract

Starch is an important quality index in potato, which contributes greatly to the taste and nutritional quality of potato. At present, the determination of starch depends on chemical analysis, which is time consuming and laborious. Thus, rapid and accurate detection of the starch content of potatoes is important. This study combined hyperspectral imaging with chemometrics to predict potato starch content. Two varieties of Kexin No.1 and Holland No.15 potatoes were used as experimental samples. Hyperspectral data were collected from three sampling sites (the top, umbilicus, and middle regions). Standard normal variate (SNV) was used for spectral preprocessing, and three different methods of competitive adaptive reweighted sampling (CARS), iterative variable subset optimization (IVSO), and the variable iterative space shrinkage approach (VISSA) were used for characteristic wavelength selection. Linear partial least‐squares regression (PLSR) and nonlinear support vector regression (SVR) models were then established. The results indicated that the sampling site has a considerable impact on the accuracy of the prediction model, and the umbilicus region with CARS‐SVR model gave best performance with correlation coefficients in calibration (Rc) of 0.9415, in prediction (Rp) of 0.9346, root mean square errors in calibration (RMSEC) of 15.9 g/kg, in prediction (RMSEP) of 17.4 g/kg, and residual predictive deviation (RPD) of 2.69. The starch content in potatoes was visualized using the best model in combination with pseudo‐color technology. Our research provides a method for the rapid and nondestructive determination of starch content in potatoes, providing a good foundation for potato quality monitoring and grading.

## Graphical abstract

In this paper, the starch content in different positions of potato was predicted and visualized.

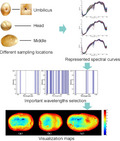

## 1 INTRODUCTION

Potatoes contain nutrients such as vitamin C, vitamin B6, folic acid, potassium, iron, and magnesium (Ji et al., 2019). With an annual global output of approximately 400 million metric tons, potatoes have become one of the four major global food crops, according to the United Nations Food and Agriculture Organization. They are mainly processed into various products, including frozen or dehydrated potatoes, potato chips, and potato starch (Rady et al., 2015). Starch content is an important quality indicator of potatoes. The starch content affects the taste of potatoes (Jiang et al., 2015), if the starch content is too high, the potatoes would be rough and hard, and if the starch content is too low, the potatoes would not be crisp. Moreover, the starch content will affect the type of potato processed products. With the popularity of science‐based diets in modern society, producers and consumers require knowledge on the starch content of potatoes to rationalize their price of potatoes and diet plans.

Conventionally, potato starch content is chemically determined, which is costly, time consuming, destructive to samples, and has high requirements on experimental skills (Chen et al., 2009). Therefore, it is very necessary to study a rapid, nondestructive, and high‐precision detection method.

Hyperspectral imaging (HSI) can simultaneously obtain two‐dimensional spatial and one‐dimensional spectral information that corresponds to internal and external features. As a powerful analytical tool, HSI is widely used in nondestructive testing (Wang et al., 2018) of fruit maturity (Chu et al., 2016; Wei et al., 2014), crop variety (Moreno et al., 2014; Williams & Kucheryavskiy, 2016), and meat quality (Khulal et al., 2016; Li et al., 2015), among other applications. Qiao (Qiao et al., 2005) created a prediction model of potato water content by using hyperspectral equipment and artificial neural networks and reported correlation coefficients between predicted and actual water content, coefficient of determination (R2) of the training set was 0.932, while that of the test set was only 0.769, with a root mean square error of 0.014. Song (Song & Wu, 2016) also used hyperspectral imaging technology to predict the moisture, starch, and dry matter of potatoes, and the results showed that hyperspectral imaging could realize the detection of various components. Jiang (Jiang et al., 2015) constructed a prediction model of potato starch content using smoothing algorithms, principal component analysis, and partial least squares (PLS) processing of hyperspectral images and obtained good predictive performance with R^2^ of 0.9031 and RMSEP of 0.5025. Sanchez et al. (2020) and Kjær et al.'s (2016) show that hyperspectral images can predict potato starch content. Therefore, hyperspectral images can predict the internal components of potatoes, and scholars have made some achievements in predicting the starch content of potatoes by using hyperspectral images, which paves the way for future scientific research, but their sampling points are concentrated in the middle regions of potatoes.

The spectral characteristics are related to the species and content of substances in the samples. Bandana (Bandana et al., 2016) found that the contents of starch, protein, and reducing sugar in the umbilicus, top and middle regions are different. Thus, the spectral information of these parts is different, which affects the prediction of starch content. Researchers usually select a random region of interest for the study, which brings uncertainty to the prediction accuracy. In addition, region mask segmentation is used as a common means, but the image processing is time consuming and does not facilitate the industrial application of this technique. Therefore, studying the effect of different sampling sites on starch prediction accuracy can optimize the best way to provide an accurate and fast sampling method. However, there is no report on the influence of research location on starch content prediction. HSI is superior to traditional near‐infrared spectroscopy due to its ability to visualize and map the content and distribution of target compounds in the sample. Such visualized images provide an important technical support for quality evaluation and grading of potatoes in industrial production lines. However, no studies have been published on the visualization of starch content in intact potatoes using HSI. The present study was intended to fill the gap of previous studies and explore the effect of different sampling sites on the prediction of starch content in intact potatoes, which has provided a reasonable sampling method. Meanwhile, the advantages of HSI are fully utilized to present the distribution of starch in intact potatoes by chemical imaging, which provides technical support for industrial grading of potatoes.

Thus, the main objectives of this study were to (1) influence of location (focusing mainly at the top, umbilicus, and middle regions) on prediction model of starch content, determine the ideal position for potato starch content detection, (2) construct starch content prediction models of full spectra and characteristic wavelength, and (3) the visual distribution of starch content in potato was obtained.

## 2 MATERIALS AND METHODS

### 2.1 Preparation of experimental samples

Kexin No.1 and Holland No.15 potatoes that appeared fresh and ripe and had no surface defects were purchased from the farmers market in Hohhot, Inner Mongolia Autonomous Region, China. Prior to the experiment, they were cleaned with water, wipe clean with absorbent paper, numbered in sequence, and kept in the dark for approximately 24 hr. In total, 96 potato samples were selected for hyperspectral data collection and chemical analysis.

### 2.2 Hyperspectral image acquisition

#### 2.2.1 HSI system

**FIGURE 1:**
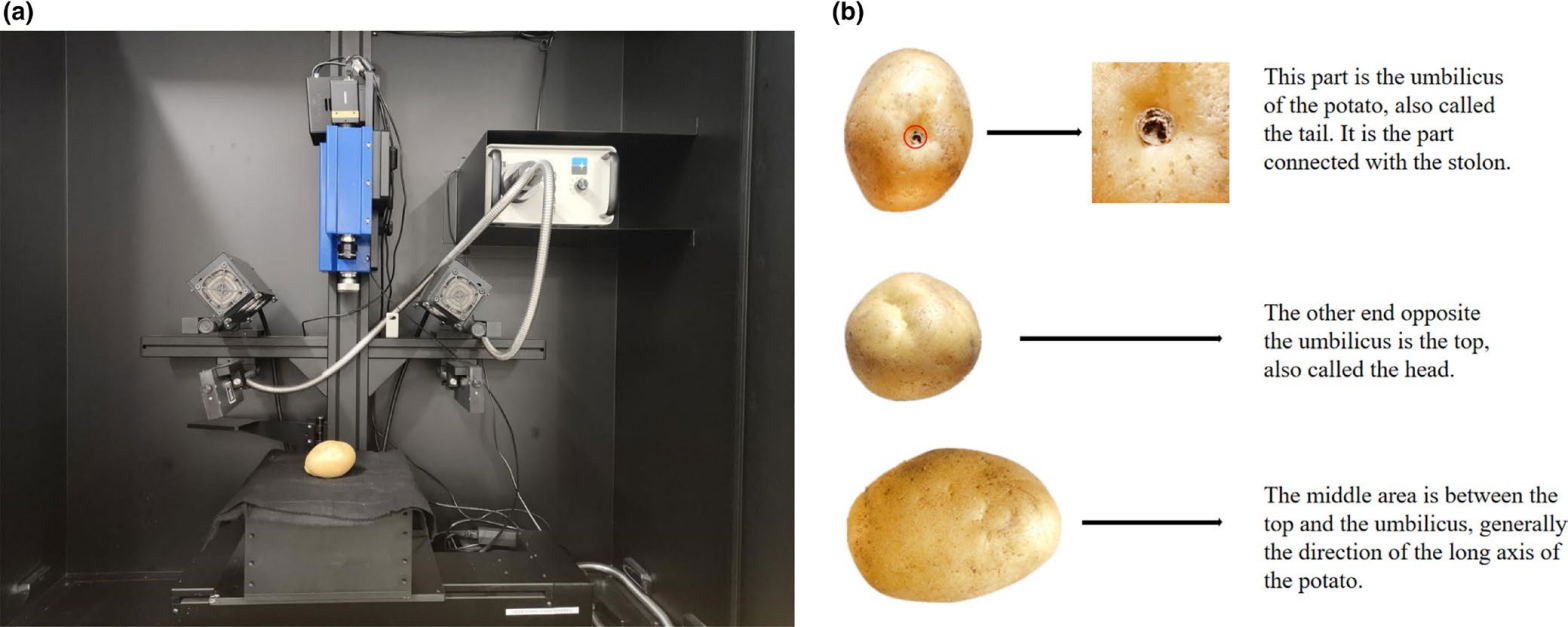
(a) Hyperspectral imaging system used in this study; (b) different sampling sites (umbilical, top, and middle area) in potato

Five‐bell optical hyperspectral imaging system is used in the experiment, which mainly includes hyperspectral image spectrometer (ImSpector V10E, Spectral Imaging Ltd, Oulu, Finland), CCD camera (IGVB1620, Imperx, USA), two 150 W halogen lamps (Type 3,900, Illuminator, Illumination Technology, USA), one DC adjustable light source (Type 2,900, Illumination Technology, USA), mobile control platform (IRCP0076‐1 COM, Taiwan, China), and computer. The spectral range of hyperspectral camera is 382–1004 nm. These components are placed in a darkroom to prevent the influence of external illumination. Before image acquisition, the system was turned on and warmed up for 30 min. Potatoes were divided into the head (top) and tail (umbilicus), the end of which was connected to the stolon, as shown in Figure 1. The middle region was the part between the top and the umbilicus. Spectral imaging information for the top, umbilicus, and middle regions of the potatoes were collected separately.

#### 2.2.2 Hyperspectral image correction

To minimize and remove dark current noise, the original images were corrected to black‐and‐white images according to the following equation (Wang et al., 2019):

(1) $$C=\frac{\text{R - B}}{\text{W - B}}\times 100\text{\textbackslash{}\%}\;$$

where C, R, B, and W are the corrected image, original image, black reference image obtained by completely covering the camera lens with the lens cover (approximate reflectivity of 0%) and white calibration image (approximate reflectivity of 99.99%), respectively.

### 2.3 Experimental method

#### 2.3.1 Chemical analysis

The starch content was determined through enzymatic hydrolysis (Nielsen & Gleason, 1945). A total of 2–5 g of the ground sample was accurately weighed and placed in a funnel with a folded filter paper. The fat was washed five times with 50 ml of ether followed by 85% ethanol to remove soluble sugars. The remainder of the sample was transferred to a 250‐mL beaker, after which the starch was heated in a boiling water bath for 15 min to gelatinize it. When the gelatinized starch was cooled to 60℃, 20 ml of amylase solution was added to it. The solution was held at 55℃ to 60℃ for 1 hr and constantly stirred. Then, one drop of hydrolysate was taken, and one drop of iodine solution was added to the solution. Then, 20 ml of amylase solution was added and hydrolyzed until the blue color of the iodine solution faded. The solution was then heated to boiling and cooled, after which it was transferred to a 250‐mL volumetric flask. Water was added to the scale, and the solution was stirred and filtered. A total of 50 ml of the hydrolysis solution was poured into a 100‐mL volumetric flask. Moreover, 5 ml of hydrochloric acid at a concentration of 6 N was added to this flask and heated in a water bath at 70℃ for 15 min. After cooling, two drops of alkali red indicator were added and neutralized with 20% sodium hydroxide solution. Water was added to the mark and stirred well. A total of 50 ml of the aforementioned solution was taken, and the reducing sugar content was determined using Felling's solution method. Finally, it was determined by reducing sugar and converted it into starch.

#### 2.3.2 Spectral data acquisition

The corrected hyperspectral images were imported into ENVI 5.3 software (ITT Visual Information Solutions, Boulder, CO, USA), and a rectangular region of 100 × 100 pixels was selected as the region of interest (ROI). The average spectra of all pixels in the ROI were extracted as the spectra of samples. The average spectra of the samples were obtained. The wavelength range, including 428 bands, was 382–1004 nm. Spectral data matrices of each of the three regions were established using Excel software.

#### 2.3.3 Pretreatment of the spectral data

In HSI, because of instrumental interference and environmental factors, noise signals appear in the original spectra. Preprocessing is crucial for eliminating unnecessary information that may complicate model establishment. In the present study, the regional data matrices were preprocessed using standard normal variate (SNV) transformation, the most widely used method for preprocessing spectral data (Dong et al., 2018). In this study, spectral data preprocessing was performed using Unscrambler x10.1 (Camo Software, Oslo, Norway).

#### 2.3.4 Characteristic wavelength selection

Hyperspectral data contain hundreds of continuous wavelengths, which is redundant and multicollinearity, which is not conducive to data processing and online application. Eliminating these redundant wavelengths and selecting the optimal variable simplify the modeling process and improve model performance. These procedures are also beneficial to online industrial application and the construction of simple, economical, efficient multispectral systems.

In the present study, an improved competitive adaptive reweighted sampling (CARS) method, iterative variable subset optimization (IVSO), and variable iterative space shrinkage approach (VISSA) were selected to extract the characteristic wavelength for the original spectra of potato top, umbilicus, and middle regions. In this study, the characteristic wavelength selection was performed according to the codes downloaded from the open‐source websites using the Matlab (Version 2014a, MathWorks, Natick, MA, USA) software.

The CARS algorithm used was simplified and improved by Li et al. (2009) based on the original CARS method, which was based on Darwin's theory of evolution. In the present study, the subset with the smallest root mean square error (RMSE) is obtained by subtracting the wavelength points with a small regression coefficient from those with a large regression coefficient in the PLS model, or the optimal variable subset, was selected using cross‐validation. In total, 50 Monte Carlo samples and 10 runs of cross‐validation were used.

IVSO is a novel algorithm proposed by Wang, Yun, Deng, Fan, and Liang (Wang et al., 2015) for selecting near‐infrared spectral features. It is based on the theory that large PLS regression (PLSR) coefficients in automatic calibration data represent important variables. In IVSO, the regression coefficients generated in a submodel are normalized to eliminate interference. In each iteration round, the regression coefficients of each variable obtained from the submodel are added to evaluate its importance level. A two‐step process of weighted binary matrix sampling (WBMS) and sequential addition is employed to gradually and competitively eliminate the noninformation variables and reduce the risk of losing important variables. Thus, IVSO has higher stability than other algorithms. In the present study, the numbers of WBMS and cross‐validation runs were 8,000 and 5, respectively.

In contrast to most methods of variable selection optimization, VISSA, proposed by Deng, Yun, Liang, and Yi (Deng et al., 2014), enables statistical evaluation of the performance of variable space at every step of the process. A weighted binary matrix sampling method is used to generate submodels. Two rules are highlighted in the optimization process: first, the variable space shrinks at each step; second, the new variable space is superior to the previous one. The present study used 1,000 binary matrix samples and 5 runs of cross‐validation.

#### 2.3.5 Establishment of the regression prediction model

In this study, the top, umbilicus, and middle regions of potato samples were subjected to PLSR and support vector machine regression (SVR) under the full spectra and characteristic wavelength spectra to determine the fit between the spectra and starch content. When PLSR and SVR models were established, the input variables are consistent, both in the full spectrum and in the simplified characteristic wavelengths. For the full spectral data, all the spectrum information is input to the prediction model, and the linear PLS and nonlinear SVR models are established, respectively. For the simplified characteristic wavelength‐based models, the characteristic wavelengths were first selected by three methods, including VISSA, CARS, and IVSO. The algorithm codes and procedures of these methods are in accordance with the literatures (Li et al., 2009; Wang et al., 2015; Deng et al., 2014), without further changes. When these characteristic wavelengths were selected, the data corresponding to each wavelength was extracted and integrated into a new spectral matrix to replace the original full spectral matrix.

PLSR is one of the most widely used linear regression algorithms (Wang et al., 2021a, 2021b) and an optimal choice for constructing a prediction model. It has the advantage of considering both matrices x (spectral data) and y (starch content). In addition, it resolves the problem of the presence of a large number of variables (including collinear variables) in the original data. PLSR analysis is used to transform the original data into several independent latent variables (LVs). To prevent overfitting or underfitting of the model, the sum of RMSE values is minimized to determine the optimal number of potential variables. In the present study, the maximum number of LVs was set to 15, and fivefold cross‐validation was used to obtain the optimal number of LVs.

When the data are nonlinear, it may be difficult for PLSR models to achieve high accuracy. SVR, another commonly used modeling algorithm, solves linear and nonlinear regression problems by using different kernel functions (Hassan et al., 2019). These functions are used to nonlinearly estimate input variables, map input variables, and establish different hyperplanes in high‐dimensional space and then establish regression models in those hyperplanes. The radial basis function (RBF) kernel is the most widely used kernel function in support vector machine regression compared to the linear and polynomial kernel functions. RBF has the advantage of reducing the computational complexity of the training algorithm and performs well under a general smoothing assumption. The grid search method and fivefold cross‐validation were used to optimize the key parameters.

The PLSR and SVR used Matlab 2014a software (MathWorks, Natick, MA, USA), PLSR modeling is contained in the libPLS_1.98 toolbox. SVR modeling was performed using the libsvm_3.1 toolbox.

## 3 RESULTS AND DISCUSSION

### 3.1 Spectral characteristics

**FIGURE 2:**
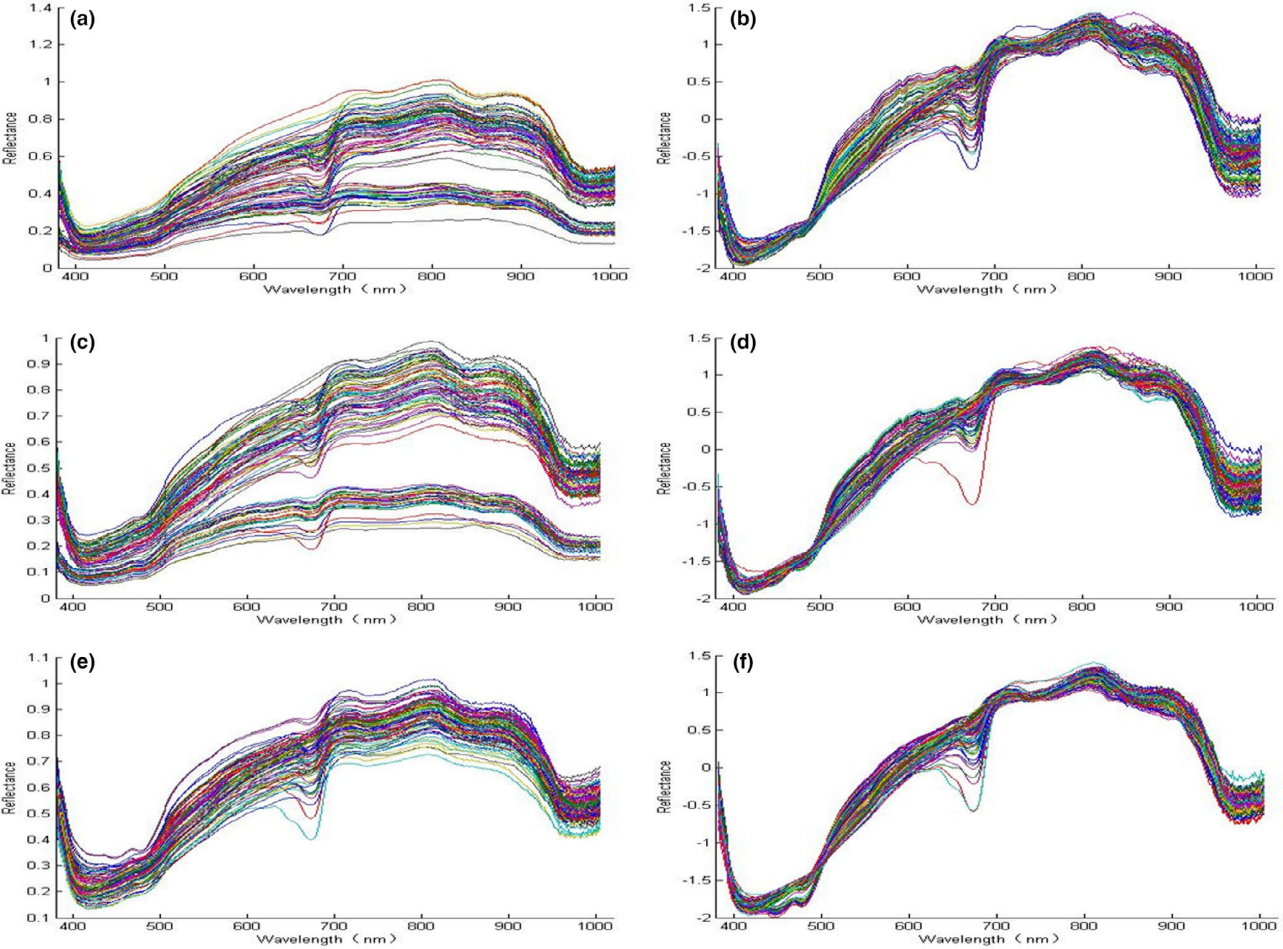
Raw and SNV preprocessed spectral curves of all potato samples. (a) raw data from top site; (b) SNV preprocessed data from top site; (c) raw data from umbilical site; (d) SNV preprocessed data from umbilical site; (e) raw data from middle site; and (f) SNV preprocessed data from middle site

By extracting the average spectral information of the ROIs, the original average spectra of the top (Figure 2(a)), umbilicus (Figure 2(c)), and middle (Figure 2(e)) of the samples were obtained. The original spectrogram indicates that the average spectral curves of all samples were similar to some extent but with different reflection intensities. Obvious separation was observed in the top and base spectral curves. The absorption value of the Kexin No.1 samples was higher than that of the Holland No.15 ones, indicating differences in the compounds between varieties, whereas those in middle regions were highly similar.

Clear absorption peaks were observed at approximately 410 nm, 680 nm, and 980nm. Slight absorption peaks appeared at approximately 750 nm and 850 nm, which were related to the tensile vibration of C–H and O–H bands in this region. The fourth C–H stretching overtone band was generated by the absorbance of glucose and fructose at 680 nm (Chen et al., 2010; Sugiyama & Junichi, 1999; Workman & Weyer, 2007). Its proximity to 750 nm may be related to the third O–H band and fourth C–H harmonic band (Jamshidi et al., 2014; Magwaza et al., 2012). The slight absorption peak at approximately 850 nm may be attributable to the third C–H overtone, which represents the absorption band of glucose and is related to the hydrocarbon group (Chen et al., 2010). There was an absorption peak near 430nm, which was considered to be carotenoid. The two varieties used in this study were yellow meat varieties with high carotenoid content (Xu et al., 2018). The bands at 980 nm may be attributable to the presence of carbohydrates and water (Farhadi et al., 2020; Kawano et al., 1992; Zhu et al., 2017).

In order to extract the characteristic wavelength and establish the model more accurately, SNV is used to preprocess the original spectra of different regions, including the SNV preprocessed spectra at the top of Figure 2(b), the SNV preprocessed spectra at the umbilical of Figure 2(d), and the SNV preprocessed spectra at the middle region of Figure 2(f).

### 3.2 Characteristic wavelength selection

Characteristic wavelengths were selected from the spectra using CARS, VISSA, and IVSO. Table 1 presents the screening results.

**TABLE 1:** Wavelength selection for starch content prediction in potato samples

Site	Method	Number	Wavelength
Top	CARS	23	382、450、478、480、481、482、632、899、903、905、908、921、924、931、943、957、975、984、987、997、1,000、1,003、1,005 nm
	VISSA	96	382、1,000、1,005、384、996、963、943、990、908、988、978、833、397、388、909、912、407、890、840、942、861、949、424、960、843、878、873、923、929、981、911、751、426、478、1,002、710、828、940、738、651、864、514、887、900、936、673、951、404、521、425、819、508、685、535、682、557、735、814、647、852、733、509、512、478、507、418、421、515、736、846、511、663、753、860、739、531、593、711、415、519、516、791、428、505、588、594、741、747、567、591、754、538、559、534、524、606 nm
	IVSO	140	484、467、751、460、744、482、470、461、415、433、464、411、747、438、742、436、439、908、443、909、809、440、468、710、481、833、1,000、480、857、759、410、444、760、705、407、457、711、707、733、485、770、764、693、903、708、489、698、487、773、772、713、822、776、692、477、741、478、837、695、806、766、824、696、997、382、691、453、701、491、769、408、406、463、401、802、440、488、704、831、738、458、699、466、732、689、450、753、851、819、404、912、808、471、719、821、905、429、511、509、702、688、1,005、958、840、996、805、422、504、899、978、739、745、426、502、508、514、761、757、512、498、775、848、966、794、1,003、417、505、507、839、828、475、474、767、495、499、720、863、515、497、854 nm
Umbilicus	CARS	20	399、489、516、519、521、522、524、525、680、682、808、903、908、911、933、936、942、961、966、996 nm
	VISSA	85	382、911、933、1,003、914、930、949、929、488、970、526、833、982、834、876、991、389、625、894、908、522、948、996、976、858、805、489、872、888、519、981、960、406、849、439、399、1,005、836、794、964、620、831、797、485、830、524、705、704、616、593、839、614、767、590、763、787、800、973、525、788、881、782、591、612、594、769、426、671、793、424、967、443、781、784、642、613、785、802、790、791、803、796、617、609、799 nm
	IVSO	259	744、733、837、485、470、742、497、484、730、482、481、498、770、840、732、495、475、487、468、727、499、736、480、729、769、767、842、478、477、843、764、745、501、488、502、726、722、739、724、763、723、489、735、474、836、747、772、460、720、766、504、738、775、494、773、849、716、839、741、505、492、845、776、451、507、761、717、834、491、473、508、779、714、456、719、457、471、509、713、778、846、781、447、511、711、760、512、453、467、454、848、710、782、449、458、833、514、450、708、759、707、515、516、831、518、784、519、466、522、521、750、389、788、524、705、525、446、526、443、461、444、851、828、787、464、785、463、438、392、528、386、529、382、748、704、532、890、882、531、790、534、407、830、536、442、535、757、911、390、538、539、852、388、751、791、756、542、891、440、544、541、396、545、435、385、888、546、896、548、881、848、410、395、558、393、433、549、876、854、555、557、551、702、552、400、554、914、559、793、872、439、894、884、885、887、432、562、594、384、561、436、564、893、794、567、565、397、875、873、593、879、568、583、581、577、571、580、570、578、574、596、575、753、902、584、585、572、587、903、591、606、590、604、855、908、588、598、603、431、701、429、600、930、899、796、597、601、415、607、612、426、897、686、609、685、929、610、870、933 nm
Middle	CARS	12	457、480、508、509、594、912、914、915、932、945、964、972 nm
	VISSA	122	915、909、945、964、908、912、382、960、982、511、1,000、988、903、482、906、507、512、505、905、649、914、504、911、657、837、572、654、508、509、651、652、973、478、942、1,002、502、544、822、648、824、936、976、484、501、981、827、716、812、972、815、642、647、814、481、803、644、811、488、818、426、435、645、825、545、793、978、436、809、531、722、723、425、536、438、802、717、485、800、985、499、529、534、756、761、759、546、431、535、711、757、541、428、831、984、498、538、696、754、433、539、713、719、730、767、532、819、727、439、432、726、769、797、805、558、821、979、994、720、729、732、557、790 nm
	IVSO	26	912、915、945、733、932、382、935、401、428、909、911、914、764、393、930、908、775、722、705、707、736、396、403、716、940、742 nm

Abbreviations: CARS, competitive adaptive reweighted sampling; IVSO, iterative variable subset optimization; VISSA, variable iterative space shrinkage approach.

**FIGURE 3:**
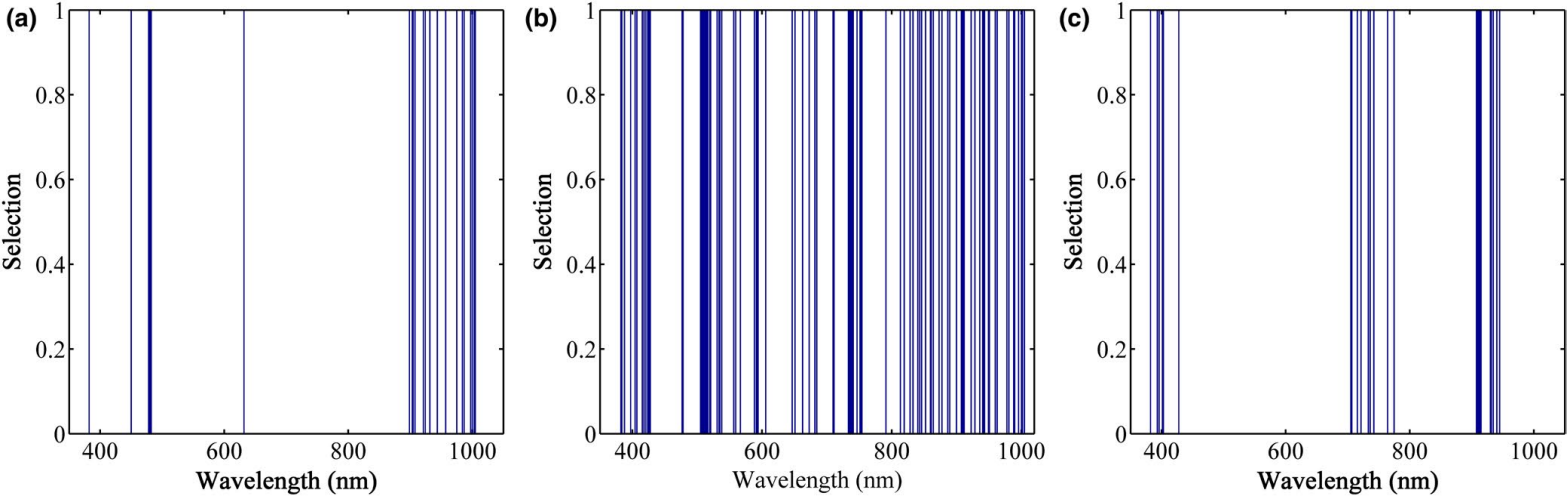
Results of characteristic wavelength selection using CARS for spectral data from (a) top, (b) umbilical, (c) and middle sites of the potatoes

It can be seen from Table 1 that in the extraction of the spectral feature information of the top, umbilicus, and middle regions of the potato, CARS performed the best of all the methods. Compared with the full spectra, the spectral variables of the top, umbilicus, and middle region are selected after the variables are selected. The numbers have been reduced by 99.95%, 99.95%, and 99.97%, respectively, indicating that CARS can simplify the model for potato starch detection. Figure 3 presents the spectral selection results on CARS. The selected characteristic wavelengths were primarily concentrated in the region of 900–1000 nm and at 450 nm. These results are almost consistent with those of Jiang (Jiang, 2017). From Figure 3, for the same variable selection method, the characteristic wavelengths selected are different for three sampling sites. The possible reason for this is that the spectra are reflected at different angles when sampled at different locations due to the spherical structure.

### 3.3 Construction of the model for predicting starch content

#### 3.3.1 Distribution of the starch content

**FIGURE 4:**
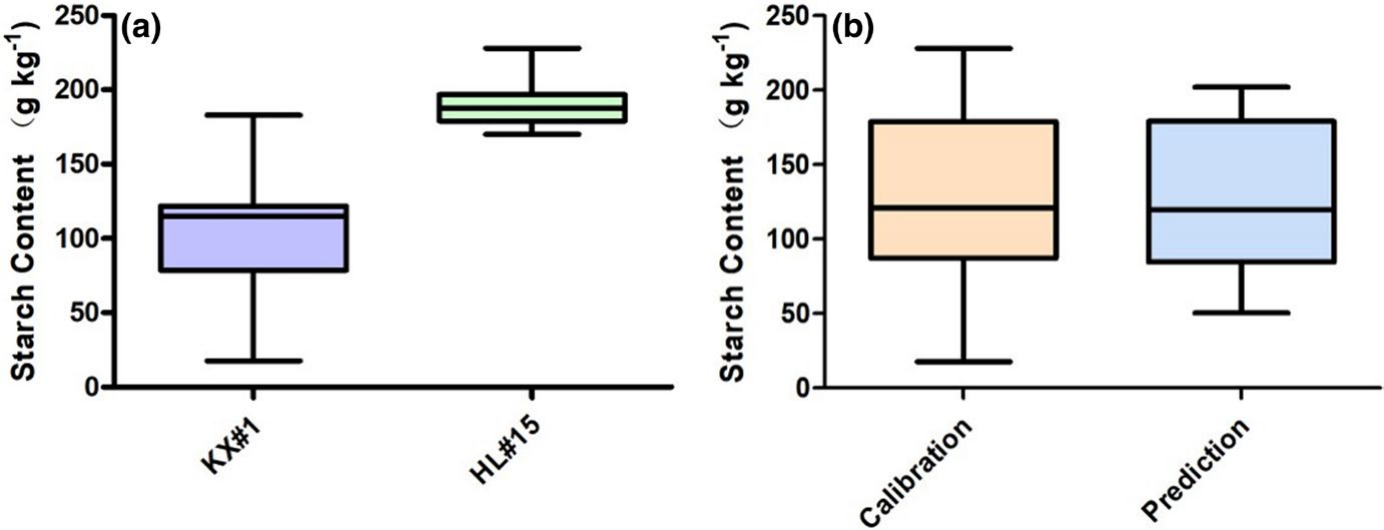
Box plots of starch content in potato samples. (a) in two different varieties; (b) in calibration and prediction sample sets

Potato tubers contain a lot of starch, which is the main source of energy for potatoes. The starch content of two potato varieties in Inner Mongolia, namely Kexin No.1 and Holland No.15 potatoes, was analyzed. Their starch content is shown in Figure 4(a). It can be seen from Figure 4(a) that the starch content of the two potatoes is quite different, and the starch content of Holland No.15 is higher than that of Kexin No.1. After the starch content test, all the potato samples were divided into a calibration set and prediction set.

The 96 potato samples were numbered consecutively and divided into calibration and prediction sets at a 2:1 ratio (64 and 32 samples). The model was corrected by the calibration set, and model robustness was tested using the prediction set. As shown in Figure 4(b), the range of starch content in the prediction set was covered by the range in the calibration set, demonstrating that the sample division was reasonable and that the prediction set could be used to test the robustness of the final model (Fan et al., 2015).

#### 3.3.2 Prediction model of potato starch content in the whole band

After preprocessing using SNV transformation, PLSR and SVR models based on the full spectra were established. The testing results are shown in Table 2.

**TABLE 2:** Results of PLSR and SVR models on full spectral data for starch content prediction in potato samples

Model		NVs	P	Calibration set		Prediction set		
				Rc	RMSEC	Rp	RMSEP	RPD
Top	PLSR	428	LVs=8	0.8881	21.8	0.8922	22.2	2.11
	SVR	428	c = 1,024, g = 0.005	0.9414	15.9	0.8988	20.8	2.25
Umbilicus	PLSR	428	LVs=2	0.8268	26.5	0.8869	22.2	2.11
	SVR	428	c = 588.134, g = 0.002	**0.9216**	**18.5**	**0.9042**	**20.1**	**2.33**
Middle	PLSR	428	LVs=5	0.8719	23.4	0.8584	24.8	1.89
	SVR	428	c = 1,024, g = 0.001	0.8938	21.2	0.8891	21.9	2.14

Abbreviations: LVs, number of latent variables; NVs, number of variables; P, parameters of models; PLSR, partial least‐squares regression; Rc, calibrated correlation coefficient; RMSEC, root mean square error of calibration; RMSEP, root mean square error of prediction; Rp, predicted correlation coefficient; RPD, residual prediction deviation; SVR, support vector machine regression.

Typical statistical parameters were used for performance evaluation of the prediction model: calibrated and predicted correlation coefficients (Rc and Rp), RMSE of calibration (RMSEC) and prediction (RMSEP), and residual prediction deviation (RPD). In general, an accurate model should have higher Rc and Rp values and lower RMSEC and RMSEP values. An RPD of <2 indicates poor prediction performance. Performance is considered to be fair when 2 < RPD <2.5 and excellent when RPD >2.5 (Cortés et al., 2019).

As indicated in Table 2, the SVR model exhibited greater stability and accuracy than the PLSR model. In addition, we also find that the same model performs worst in the middle area model, and the middle area is the frequently used sampling point. The SVR model achieved the best performance for the umbilicus. The corresponding numbers are indicated in bold in Table 2.

#### 3.3.3 Prediction model of potato starch content in characteristic bands

To simplify the modeling process and improve model performance, the characteristic wavelengths selected using CARS, VISSA, and IVSO were used to replace the full spectra variables to establish a calibration model after spectral preprocessing. Table 3 presents the modeling effects.

**TABLE 3:** Results of PLSR and SVR models on characteristic wavelengths for starch content prediction in potato samples

Site		PLSR						SVR					
		LVs	Rc	RMSEC	Rp	RMSEP	RPD	c & g	Rc	RMSEC	Rp	RMSEP	RPD
Top	CARS	4	0.9277	17.7	0.9008	20.5	2.29	337.794, 0.047	**0.9361**	**16.8**	**0.9284**	**17.7**	**2.65**
	VISSA	10	0.8992	21.1	0.8181	29.2	1.61	1,024, 0.016	0.9416	16.1	0.8916	21.5	2.18
	IVSO	8	0.9088	19.8	0.8907	21.7	2.16	1,024, 0.016	0.9439	15.8	0.9108	19.6	2.39
Umbilicus	CARS	6	0.9309	17.8	0.9077	19.9	2.36	1,024, 0.047	**0.9415**	**15.9**	**0.9346**	**17.4**	**2.69**
	VISSA	9	0.9159	19.0	0.8803	22.7	2.06	1,024, 0.027	0.9591	13.4	0.9208	18.4	2.55
	IVSO	11	0.8821	22.5	0.9049	20.5	2.29	1,024, 0.002	0.9076	19.8	0.9211	18.5	2.53
Middle	CARS	5	0.9067	20.1	0.8861	22.1	2.13	588.1336, 0.082	0.9297	17.7	0.9054	20.5	2.29
	VISSA	5	0.8954	21.3	0.8521	25.2	1.87	1,024, 0.016	0.9305	17.4	0.8931	21.5	2.18
	IVSO	**5**	**0.9098**	**19.8**	**0.9037**	**20.3**	**2.32**	111.431, 0.25	0.9018	20.9	0.8455	25.6	1.83

As indicated in Table 3 of the models established for the characteristic wavelength in the top, CARS‐SVR model achieved the best performance (Rc =0.9361, Rp =0.9284, RMSEC =16.8 g/kg, RMSEP =17.7 g/kg, RPD =2.65). Of the models established for the characteristic wavelength in the umbilicus, CARS‐SVR model achieved the best performance (Rc =0.9415, Rp =0.9346, RMSEC =15.9 g/kg, RMSEP =17.4 g/kg, RPD =2.69). Of the models established for the characteristic wavelength in the middle region, IVSO‐PLSR had the best modeling effect (Rc =0.9098, Rp =0.9037, RMSEC =19.8 g/kg, RMSEP =20.3 g/kg, RPD =2.32).

### 3.4 Model comparison and discussion

In this study, the original spectra of potato umbilicus, top, and middle region were collected, and the original spectra were preprocessed, and the prediction model of starch content under full spectrum and characteristic wavelength was established.

When we use the same prediction model at different sampling locations, we find that the detection results of the models are different and have a big gap, which shows that the top, umbilicus, and middle region of potato will greatly affect the establishment of the model. Moreover, whether it is the starch content model established by full spectrum or the starch model established by characteristic wavelength, the model established in the middle region of potato is relatively poor, and the model established in the umbilicus has the best performance, which is different from the previous scholars' research focus, which shows that in future research, in order to make the model achieve better results, the selection of sampling points is also an important factor to be considered.

The PLSR models under full spectrum and characteristic wavelength were established in the top, umbilical, and middle area, respectively. By comparison, we found that CARS‐PLSR and IVSO‐PLSR models at three locations were better than those established by full spectrum, while VISSA‐PLSR models were worse than those established by full spectrum, which indicated that the characteristic wavelength extracted by CARS and IVSO was effective, and the correlation between the characteristic information extracted by VISSA and starch content was low, CARS‐PLSR model established in umbilical region has the best performance, among which Rc=0.9309, RMSEC=17.8, Rp=0.9077, RMSEP=19.9, RPD=2.36.SVR models under full spectrum and characteristic wavelength are established in the top, umbilical, and middle regions, respectively. We can see that the models established by characteristic wavelength in umbilical have better performance than the models established by full spectrum, which shows that it is necessary to screen characteristic wavelength in umbilical, while VISSA‐SVR at top has worse performance than the models established by full spectrum. Similar to this result, the IVSO‐SVR model in the middle region is also worse than the model established by the full spectrum. The CARS‐SVR model in the umbilical performs best, in which Rc=0.9415,RMSEC=15.9, Rp=0.9346, RMSEP=17.4, RPD=2.69, this further shows that although VISSA and IVSO are relatively new algorithms and have not been used in potato hyperspectral research, through analysis and comparison, it is found that VISSA feature wavelength extraction algorithm is not accurate enough for potato starch content, and IVSO feature wavelength extraction algorithm is relatively feasible, while CARS is a method worth considering, which not only extracts fewer wavelengths but also is more accurate.

HSI has been applied to quantitative research on potatoes in recent years. Research on potato starch has mainly focused on the middle region of the potato. Jiang (Jiang, 2017) detected potato starch content using HSI coupled with a Random‐Frog–PLS model and obtained the following results: Rc^2^ = 0.8514, RMSEC =0.3259, Rp^2^ = 0.8348, and RMSEP =0.2906. Our CARS‐SVR results (Rc^2^ = 0.8864, Rp^2^ = 0.8768, RMSEC =15.9 g/kg, RMSEP =17.4 g/kg, RPD =2.69) indicate that our model achieved better prediction accuracy. Li (Li, 2018) used HSI to detect potato starch content and reported that the Random‐Frog–PLS model had the best predictive effect (Rc^2^ = 0.8624, RMSEC =0.3249, Rp^2^ = 0.8343, RMSEP =0.2926). The characteristic wavelengths constituted approximately 6% of the total wavelength range. The prediction accuracy of our model was considerably better. In addition, our characteristic wavelengths constituted approximately 1% of the total wavelength range, indicating that our characteristic wavelength extraction algorithm was more precise. Due to the spherical structure of intact potatoes, the reflection of the spectrum is different at different parts of the potato surface. Researchers usually choose a random region of interest to study, which introduces uncertainty in the accuracy of the prediction. Region mask segmentation is commonly used, but the image processing is time consuming and not conducive to the industrial application of this technique. Therefore, studying the effect of different sampling sites on the accuracy of starch prediction can optimize the best way to provide accurate and fast sampling methods. In this study, the effect of different sampling sites on the prediction accuracy of starch content in intact potatoes was explored for the first time, and the best sampling site was optimized. In addition, previous studies have not imaged the distribution of starch in potatoes. In this study, the advantages of HSI were fully exploited, and the starch content and distribution in intact potatoes were clearly mapped.

### 3.5 Visualized distribution of starch

**FIGURE 5:**
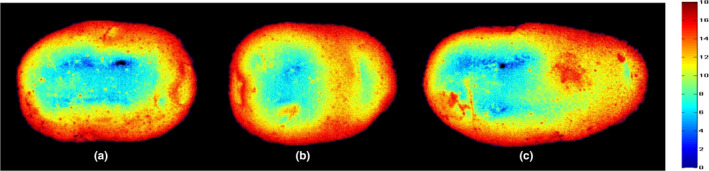
Visualization maps of starch content in potato samples

It is difficult to visually see the distribution of potato starch content with naked eyes. After we get the best model, we use the best model and pseudo‐color technology to realize the visual distribution of potato starch content through MATLAB program, the value of each pixel at the important wavelength is extracted and introduced into the constructed model to determine the starch content (Kandpal et al., 2013), but this research has not been reported. Figure 5 shows the intuitive distribution of potato starch content. A dark to light color indicates a change in starch content from low to high. The mass fractions of (a), (b), and (c) are 12.5%, 13.1%, and 13.6%, respectively, which are determined by chemical methods.

It can be seen from the visual images that the starch content of samples a, b, and c gradually increases, in this way, we can clearly observe the difference in the content of different potatoes. However, such visualizations of the traditional visible‐near‐infrared spectrum are not possible. To sum up, the chemical image of potato starch content was obtained by hyperspectral imaging, which provided a new method for rapid evaluation of potato quality and storage and preservation.

## 4 CONCLUSION

In this study, we used the visible‐near‐infrared hyperspectral equipment to collect the spectral and image information of the top, navel, and middle region of potato, and preprocessed the collected spectral information by SNV. After preprocessing, we established the spectral model of full spectrum and characteristic wavelength. The whole spectral model under characteristic wavelength has been improved, which shows the importance of selecting characteristic wavelength. After comparing the models established in the top, navel, and middle area, we found that the performance of the models varies greatly due to different sampling points, and the CARS‐SVR model established in the navel performs best, RC =0.9415, RMSEC =15.9, RP =0.9346, RMSEP =17.4, RPD =2.69. This also shows that the traditional method of only focusing on improving the model by algorithm is somewhat single, and we should consider not only the suitability of the algorithm but also the selection of sampling points in the later stage. After that, we also established the visual distribution of potato starch content by using the best model, which is of great help to the quality grading and quality supervision of potatoes in the future. In the future study, the way how to eliminate the interference of the spherical structure on the spectral signal will be explored, and more accurate and feasible prediction models will be provided.

## CONFLICTS OF INTEREST

The authors declare that there is no conflict of interest regarding the publication of this study.

## Data Availability

The data that support the findings of this study are available on request from the corresponding author. The data are not publicly available due to privacy or ethical restrictions.
